# Maternal supply of methionine during late-pregnancy enhances rate of Holstein calf development in utero and postnatal growth to a greater extent than colostrum source

**DOI:** 10.1186/s40104-018-0298-1

**Published:** 2018-11-23

**Authors:** Abdulrahman S. Alharthi, Fernanda Batistel, Mohamed K. Abdelmegeid, Gustavo Lascano, Claudia Parys, Ariane Helmbrecht, Erminio Trevisi, Juan J. Loor

**Affiliations:** 1 Mammalian NutriPhysioGenomics, Department of Animal Sciences and Division of Nutritional Sciences, University of Illinois, Urbana, IL 61801 USA; 2 Department of Animal, Dairy and Veterinary Sciences, Utah State University, Logan, UT 84322 USA; 3 Department of Animal Medicine, Faculty of Veterinary Medicine, Kafrelsheikh University, Kafrelsheikh, 33516 Egypt; 4 Department of Animal and Veterinary Science, Clemson University, Clemson, SC USA; 5 Evonik Nutrition & Care GmbH, Wolfgang, Hanau, Germany; 6 Department of Animal Sciences, Food and Nutrition Faculty of Agriculture, Food and Environmental Science, Università Cattolica del Sacro Cuore, 29122 Piacenza, Italy

**Keywords:** Epigenetics, Metabolism, Methyl donors, Nutritional programming

## Abstract

**Background:**

Pregnancy and early life are critical periods of plasticity during which the fetus and neonate may be influenced by environmental factors such as nutrition. Maternal methionine (Met) supply in non-ruminants during pregnancy can affect offspring development and growth. Thus, the objective of this study was to investigate if increasing Met supply during late-pregnancy affects developmental parameters of the calf at birth and if either maternal Met or colostrum from Met-fed cows alters calf growth. Calves born to Holstein cows individually-fed a basal control [CON; 1.47 Mcal/kg dry matter (DM) and 15.3% crude protein] diet with no added Met or CON plus ethylcellulose rumen-protected Met (MET; Mepron® at 0.09% of diet DM; Evonik Nutrition & Care GmbH, Germany) during the last 28 ± 2 d of pregnancy were used. A total of 39 calves were in CON (*n* = 22 bulls, 17 heifers) and 42 in MET (*n* = 20 bulls, 22 heifers). At birth, calves were randomly allocated considering dam treatment and colostrum as follows: 1) calves from CON cows and colostrum from CON cows (*n* = 21); 2) calves from CON cows and colostrum from MET cows (*n* = 18); 3) calves from MET cows and colostrum from MET cows (*n* = 22); and 4) calves from MET cows and colostrum from CON cows (*n* = 20). All calves were housed, managed, and fed individually during the first 9 wk of life.

**Results:**

Despite greater daily DM intake pre-partum in cows fed MET (15.7 vs. 14.4 ± 0.12 kg/d, *P* < 0.05), colostrum quality and quantity were not affected by maternal diet. At birth, MET calves had greater (*P* ≤ 0.05) body weight (BW, 44.1 vs. 42.1 ± 0.70 kg), hip height (HH, 81.3 vs. 79.6 ± 0.53 cm) and wither height (WH, 77.8 vs. 75.9 ± 0.47 cm). In contrast, concentrations of His, Lys, and Asn in plasma were lower (*P* ≤ 0.05) in MET calves. Regardless of colostrum source, the greater BW, HH, and WH in MET calves at birth persisted through 9 wk of age resulting in average responses of + 3.1 kg BW, + 1.9 cm HH, and + 1.8 cm WH compared with CON. Average daily gain during the 9 wk was (*P* < 0.05) 0.72 ± 0.02 kg/d in MET compared with 0.67 ± 0.02 kg/d in CON calves. Respiratory scores were normal and did not differ (*P* > 0.05) due to maternal Met supply or colostrum source. However, fecal scores tended to be lower (*P* ≤ 0.10) in MET calves regardless of colostrum source.

**Conclusions:**

Increasing the maternal supply of MET during late-pregnancy enhanced growth in utero as well as during the pre-weaning and early post-weaning periods. Although the ~ 1 kg/d greater DM intake during the last 2–3 wk prior to parturition could explain a portion of the 2 kg extra body mass of MET calves at birth, other mechanisms potentially encompassing nutrient assimilation efficiency likely played a role. Assessing the exact mechanisms sensitive to supply of Met or total amino acid supply during the latter stages of growth in utero merit further research.

**Electronic supplementary material:**

The online version of this article (10.1186/s40104-018-0298-1) contains supplementary material, which is available to authorized users.

## Background

The maternal diet during pregnancy is an important factor that can affect offspring health and performance [1]. Around parturition time, the increased demand for nutrients to sustain fetal growth and lactation expose animals to negative energy and amino acid (AA) balance. As such, improving the essential AA (EAA) profile and particularly Lys and Met (the most-limiting AA for milk synthesis [2]) in the metabolizable protein (MP) through nutrition during the periparturient period has historically been an important area of research [3]. The recognition that Met metabolism can generate antioxidants (taurine, glutathione) and S-adenosylmethionine (SAM) [4, 5] also shifted the research focus on better understanding its “functional role” in the context of health, immune function, and performance [5–9].

Despite abundant evidence in non-ruminants, few studies have addressed the role of nutrient manipulations during late-gestation and their influence in fetal and postnatal development of the bovine offspring [6, 10]. “Fetal programming” is an important concept that seeks to explain the effect of maternal nutrition on long-term offspring growth and health [11, 12]. Recent work with beef cattle has revealed that maternal nutrient restriction during late-gestation reduces postnatal calf birth weight [13]. In contrast, protein supplementation during late-pregnancy tends to increase calf body weight (BW) at weaning and increase pre-breeding BW compared with unsupplemented diets [14]. Clearly, these data underscore the role of maternal nutrition on offspring growth and performance in ruminants.

Amino acids play important roles in regulating metabolism, growth, and development [15]. Evidence indicates that AA can regulate gene expression and different metabolic pathways to improve growth, health, and reproduction [16]. Maternal dietary methyl donors, e.g. Met, folic acid, and betaine, are essential nutrients during pregnancy and provide methyl groups that serve as precursors of SAM which is involved in methylation of DNA and can alter gene transcription [17, 18]. A recent study reported that supplementation of pregnant cows with rumen-protected Met (RPM) in late-pregnancy altered the expression of genes associated with gluconeogenesis, fatty acid oxidation, insulin signaling pathway, and inflammatory response in the liver of neonatal calves [19]. Those findings provided some evidence that enhancing the maternal supply of Met can cause changes related to maturation of key biologic pathways in the liver. Although no differences in growth and health parameters were detected in that study, it is likely that the number of calves used (20 per treatment) was insufficient to generate statistical inferences in those types of parameters that inherently have greater variation.

Our general hypothesis was that enhancing the supply of Met during late-pregnancy, besides benefitting cows [9, 20], would affect developmental parameters in calves at birth and subsequent growth. Thus, the specific objectives of the present study were to determine if greater supply of Met during the last 28 d of pregnancy would affect development and neonatal growth either through an utero-placental effect during pregnancy (e.g., greater maternal-fetus nutrient transport) or through colostrum (e.g., higher immunoglobulin and nutrient content).

## Methods

All the procedures for this study were conducted in accordance with a protocol approved by the Institutional Animal Care and Use Committee (IACUC) of the University of Illinois (protocol # 14270).

### Experimental design and treatments

Calves in the present study were from Holstein cows (3.22 ± 0.20 lactations) randomly assigned to receive a basal control (CON) close-up diet (from −( 28 ± 2) d to parturition) [*n* = 39; 1.47 Mcal/kg dry matter (DM) and 15.3% crude protein (CP)] with no added RPM or CON plus ethyl cellulose RPM (MET, *n* = 42; Mepron®, Evonik Nutrition & Care GmbH, Germany) diet composition is available in Additional file 1: Table S1. During the preliminary period from − 45 to − 29 d relative to parturition all cows received a common early-dry period “far-off” diet (1.33 Mcal/kg of DM and 13.9% CP) with no RPM. Cows were individually-fed using Calan gates (American Calan Inc., Northwood, NH). The Met product was offered at a rate of 0.09% of diet DM. This supply of Met was based on experiments demonstrating a benefit of achieving a Lys:Met ratio close to 2.8:1 in terms of production performance and health [6, 9]. Mepron® is a commercial rumen-protected source of DL-Met that resists ruminal degradation through an ethyl-cellulose film coating. Pellets measure 1.8 ± 3 mm and contain 85% *DL*-Met. Mepron® contains an equimolar mixture of the *D*- and *L*-isomers, and the dairy cow transforms a minimum of (75 ± 3)% of the ingested D-Met into *L*- Met [21]. The intestinal digestibility coefficient of Mepron is 90% [22] and its ruminal bypass value is 80% [23]; therefore, per 10 g of Mepron®, the cows received 6.1 g of Met available for absorption. Metabolizable protein (MP) and post-ruminal Lys and Met in far-off and close-up diets is reported in Additional file 1: Table S2.

After collecting measurements at birth, calves were randomly allocated considering dam treatment and colostrum as follows: 1) calves from CON cows and colostrum from CON cows (CC, *n* = 21); 2) calves from CON cows and colostrum from MET cows (CM, *n* = 18); 3) calves from MET cows and colostrum from MET cows (MM, *n* = 22); and 4) calves from MET cows and colostrum from CON cows (MC, *n* = 20).

### Colostrum handling, sampling, and management

After parturition, calves were removed from their dams immediately and cows were milked in the parlor where the volume of colostrum was recorded, and immunoglobulin G (IgG) content estimated based on specific gravity with a bovine colostrometer (Nasco, Fort Atkinson, WI; Cat. No. C10978N). Twenty mL of colostrum was collected from each dam and stored at − 20 °C within 30 min after collection and used for long chain fatty acid (LCFA) analysis (Clemson University, Clemson, SC, USA) and free AA concentration (Additional file 1: Table S3). Calf starter and milk replacer were sampled weekly and composed monthly for AA analysis (AOAC International, 1995) (Additional file 1: Table S4). Analysis of LCFA was performed in isolated fat converted to methyl esters by direct transesterification as described previously [24] (Additional file 1: Table S5).

At birth, body weight (BW), hip and wither height (HH, WH), hip width (HW) and body length were measured standing by at least three people. At subsequent time points, only two people handled the calves to collect these measurements. Blood samples from the jugular vein (20-gauge BD Vacutainer® needles and lithium-heparin anticoagulant, Franklyn Lakes, NJ, USA) were taken prior to feeding colostrum, followed by disinfection of the navel with a 7% tincture of iodine solution (First Priority Inc., Elgin, IL), and vaccination with TSV II (Pfizer Inc., New York, NY) via nostril application.

Calves were offered 3.8 L of first milking colostrum once within 6 h after birth, and if colostrum intake had not reached the 3.8 L required within that time-frame they were force-fed via esophageal tube to ensure all calves consumed the same amount of colostrum. If the cow produced more than 3.8 L, the excess colostrum was immediately stored at − 20 °C without preservatives. This extra colostrum was used as needed to feed other calves either in the same maternal group (CC and MM) or in a different one (CM and MC) (Table 2). Thus, throughout the study there was extra colostrum from cows in the CON and MET group available.

Per standard operating procedures, calves were housed in individual outdoor hutches bedded with straw, and fed twice daily (07:00 and 18:00 h) with a milk replacer (Advance Excelerate, Milk Specialties, Carpentersville, IL; 28.5% CP, 15% fat) until 35 d of age. At this point they were then switched to once a day feeding at 07:00 h until weaning (42 d of age). From 1 to 10 d of age, calves received 4.54 kg/d of milk replacer, from 11 to 20 d of age 5.90 kg/d, from 21 to 35 d of age 7.26 kg/d. From 36 to 42 d of age calves received 3.63 kg/d in a single feeding. From 1 d until 56 d of life calves had ad libitum access to a starter grain mix [19.9% CP, 13.5% neutral detergent fiber (NDF)] at 08:00 h. Growth performance including body weight, hip height, wither height and hip width was recorded for each calf once a week. Fecal score (scale 1–4) based on appearance was evaluated for each calf as: 1 = firm well formed; 2 = soft, pudding like; 3 = runny, pancake batter; and 4 = liquid, splatters. Respiratory score (scale 1–5) was recorded for each calf as: 1 = Normal; 2 = Runny rose; 3 = Heavy breathing; 4 = Cough moist; 5 = Cough dry [25]. Rectal temperature and starter intake were recorded daily for each calf until 56 d of life.

### Calf blood plasma biomarkers of metabolism

Blood samples were collected from the jugular vein using 20-gauge BD Vacutainer needles and lithium-heparin anticoagulant (Becton Dickinson, Franklin Lakes, NJ) at birth, 2, 7, 21, 42 and 50 d of age. Per IACUC guidelines, the same 14 calves from cows that received the CON and MET diet (*n* = 6 bulls, 8 heifers in CON or MET) were used for blood biomarker and AA analyses throughout the study (birth and subsequent times). Final breakout of calves in each of the combinations of maternal diet and colostrum type was *n* = 7 for the CC, CM, MC, and MM groups. Samples were analyzed for cholesterol (Cat. No. 0018250540), creatinine (Cat. No. 0018255540), urea (Cat. No. 0018255440), and glucose (Cat. No. 0018250840) using the IL Test purchased from Instrumentation Laboratory Spa (Werfen Co., Milan, Italy) in the ILAB 600 clinical auto-analyzer (Instrumentation Laboratory, Lexington, MA). Free fatty acids (NEFA) and hydroxybutyric acid (BHBA) were measured using kits from Wako Chemicals and Randox Laboratories Ltd., respectively [19, 20].

### Amino acid analysis

Plasma, de-fatted colostrum (Additional file 1: Table S2), and starter (Additional file 1: Table S3) AA analysis was performed according to established protocols [26]*.* The starter and milk replacer samples were obtained weekly during the first 6 wk of age. Briefly, samples were first oxidized at 0 °C for 16 h with performic acid to allow for subsequent quantification of Met and Cys. Excess performic acid was removed with an incubation with sodium sulphite for 30 min in an ice bath. Hydrolysis was then performed with hydrochloric acid at 110 °C for 24 h. A Biochrom 30+ (Biochrom Ltd., Cambridge, UK) amino acid analyzer was used for amino acid profiling.

### Statistical analysis

For the analysis at 0 d, sex was included in the model for completeness and any significant interaction between maternal diet and sex was included when significant. The calf growth data and starter intake after receiving colostrum were analyzed according to the following general model:

$$ {y}_{ijklm}=\mu +{B}_i+{M}_j+{C}_k+{MC}_{jk}+{T}_l+{S}_m+{TM}_{jl}+{TC}_{kl}+{\varepsilon}_{ijklm} $$

Where *Y*_*ijklm*_ the dependent, continuous variable; *μ* the overall mean; *B*_*i*_ is the random effect of calf (maternal diet); the fixed effects in the model includes maternal diet (*M*_*j*_, *j*= CON or MET), colostrum source (*C*_*k*,_ *k* = CON or MET), Time (*T*_*l*_), sex (*S*_*m*_, *m* = bull or heifer), and interactions. *ε*_*ijklm*_ is the residual error. The covariance structure of the repeated measurements was AR (1). Concentrations of all blood biomarkers and amino acids in postnatal colostrum samples were analyzed using the same model for growth data without including the sex of the calf in the model. This was deemed appropriate because of the limited sample size per combination of treatment and sex. The covariance structure of the repeated measurements was SP (POW).Individual daily DM intake, weekly body condition score (BCS), and weekly BW prior to calving also were analyzed. All data were analyzed using PROC MIXED of SAS (SAS Institute Inc., Cary, NC). The cow performance data prior to parturition was analyzed using a similar model including maternal diet and time. The Kenward-Roger statement was used for computing the denominator degrees of freedom for all data.. When interactions were significant, least square means separation between and within time points was performed using the PDIFF statement with Tukey adjustment. Amino acid profiles of fat-free colostrum were analyzed using the MIXED procedure without repeated measures. The AA profiles, ammonia, and CP content of starter and milk replacer were not analyzed statistically. Data were assessed for normality of distribution using the Shapiro-Wilk test. When the normality assumption was rejected, data were log-transformed before statistical analysis and log-back transformed after analysis. Statistical differences were declared significant at *P* ≤ 0.05 and tendencies at *P* ≤ 0.10.

## Results

### Cow performance

Cows fed MET had greater (*P* < 0.01) DMI (data not shown), consuming during the last 14 d of pregnancy on average 15.7 ± 0.12 kg/d compared with 14.4 ± 0.12 kg/d in cows fed CON. There were no differences (*P* ≥ 0.73) between treatments for BW (783 vs. 782 ± 15.9 kg) and BCS (3.72 vs. 3.71 ± 0.08) during the last 28 d of pregnancy.

### Growth performance at birth and during the first 9 wk of life

At birth, calves born to dams offered MET had greater BW (*P* = 0.04(, HH (*P* = 0.02(, and WH (*P* < 0.01) (Table 1). However, HW, and body length were not affected by maternal diet (*P* > 0.10).

**Table 1:** Developmental parameters and blood biomarkers at birth in calves born to cows offered a control diet (CON, *n* = 39) or CON supplemented with ethyl-cellulose rumen-protected Met (MET, *n* = 42; Mepron® at 0.09% of diet DM; Evonik Nutrition & Care GmbH, Germany) during the last 28 d of pregnancy

Item	Maternal diet		Sex			*P* value^1^		
	CON	MET	Bull	Heifer	SEM	M	S	M × S
Body weight, kg	42.1^b^	44.1^a^	44.9^a^	41.3^b^	0.70	0.04	< 0.01	–
Hip height, cm	79.6^b^	81.3^a^	81.0	79.8	0.53	0.02	0.11	–
Hip width, cm	16.3^b^	16.5^ab^	16.7^a^	16.1^b^	0.24	0.74	0.05	0.03
Wither height, cm	75.9^b^	77.8^a^	77.1	76.6	0.47	< 0.01	0.38	–
Body length, cm	110	112	112^a^	109^b^	1.0	0.30	0.04	–
Blood biomarkers, mmol/L								
Glucose	4.54	4.85	4.82	4.57	0.68	0.72	0.78	–
Cholesterol	0.59	0.67	0.64	0.62	0.03	0.09	0.69	–
NEFA	0.94	1.20	1.03	1.11	0.10	0.07	0.55	–
Urea	5.63	6.31	6.47	5.47	0.39	0.19	0.06	–
BHBA	0.07	0.05	0.06	0.06	0.01	0.11	0.48	–
Creatinine	206	225	197	234	17.0	0.42	0.11	–
Amino acids, μmol/L								
His	8.81^a^	6.51^b^	7.59	7.73	0.61	0.01	0.86	–
Ile	3.95	3.92	4.78^a^	3.08^b^	0.51	0.97	0.02	–
Leu	7.51	6.74	8.43^a^	5.82^b^	0.80	0.47	0.02	–
Lys	3.49^a^	2.54^b^	3.37	2.66	0.35	0.05	0.14	–
Met	2.41	1.80	2.21	2.01	0.24	0.06	0.54	–
Phe	4.63	4.20	4.40	4.44	0.32	0.30	0.93	–
Thr	4.72	3.69	4.66	3.75	0.66	0.24	0.30	–
Val	14.22	13.11	15.60^a^	11.74^b^	1.11	0.45	0.01	–
Ala	69.71	50.40	70.81^a^	49.30^b^	7.79	0.07	0.05	–
Arg	6.79	6.29	7.01	6.07	0.55	0.49	0.21	–
Asn	2.10^a^	1.60^b^	2.08	1.62	0.18	0.04	0.06	–
Asp	1.00	1.06	1.09	0.96	0.11	0.63	0.35	–
Glu	3.04	3.37	3.68^a^	2.73^b^	0.26	0.33	0.01	–
Gln	43.35	37.60	45.24	36.03	3.95	0.22	0.06	–
Gly	46.18	44.84	47.75	43.27	3.66	0.78	0.36	–
Pro	10.87	10.96	12.14^a^	9.69^b^	0.92	0.94	0.05	–
Ser	7.30	7.52	8.57^a^	6.25^b^	0.80	0.84	0.04	–
Tau	2.51	2.39	2.65	2.25	0.37	0.81	0.42	–
Tyr	3.40	2.78	2.96	3.22	0.25	0.08	0.44	–
Orn	0.26	0.22	0.27	0.21	0.02	0.18	0.06	–
3-methyl-His	0.35	0.31	0.32	0.33	0.04	0.48	0.79	–

^1^*M*, maternal diet effect; *S*, bull or heifer effect

^a,b^Means on the same row differ (*P* ≤ 0.05).

During wk 1 through wk 9, a main effect (*P* ≤ 0.05) of maternal diet was detected for BW, HH, WH, and average daily gain (ADG) (Table 2). Calves born to cows fed MET had greater BW (62.4 kg vs. 59.3 ± 1.9 kg), HH (88.8 cm vs. 86.9 ± 0.68 cm), WH (84.5 cm vs. 82.7 ± 0.67 cm), ADG (0.72 kg/d vs. 0.67 ± 0.02 kg/d), and tended (*P* ≤ 0.10) to have lower fecal score (1.71 vs. 1.83 ± 0.09 out of 4). The differences between treatments for HH and WH were evident at wk 1 of age and continued over time. Despite a lack of difference in daily starter intake (Table 2), clear differences in BW were evident as early as wk 4 of age.

**Table 2:** Weekly growth parameters (1–9 wk of age), daily starter intake and average daily gain (1–56 d of age) in calves born to Holstein cows offered a control diet (CON) or CON supplemented with ethyl-cellulose rumen-protected Met (Mepron® at 0.09% of diet DM; Evonik Nutrition & Care GmbH, Germany) during the last 28 d of pregnancy. A subset of calves born to CON cows received CON colostrum (CC, *n* = 21) or Met colostrum (CM, *n* = 18). Similarly, a subset of calves born to Met cows received Met colostrum (MM, *n* = 22) or CON colostrum (MC, *n* = 20). The interactions of maternal diet × sex or colostrum × sex were not signiciant (*P* > 0.05). Longitudinal means for body weight, hip height, wither height, and starter intake in calves born to CON cows or Met cows are reported in Additional file 1: Figure S1

Item	Maternal diet		Colostrum type			*P* value^1^						
	CON	MET	CON	MET	SEM	M	C	T	M × C	Sex	M × T	C × T
Body weight, kg	59.3^b^	62.4^a^	61.1	60.5	1.9	0.02	0.66	< 0.01	0.77	0.01	0.31	0.41
Hip height, cm	86.9^b^	88.8^a^	87.5	88.1	0.68	< 0.01	0.34	< 0.01	0.40	0.25	0.80	0.18
Hip width, cm	20.3	20.6	20.3	20.6	0.32	0.26	0.17	< 0.01	0.78	< 0.01	0.49	0.24
Wither height, cm	82.7^b^	84.5^a^	83.3	83.9	0.67	< 0.01	0.31	< 0.01	0.99	0.37	0.53	0.11
Body length, cm	126	128	127	126	1.01	0.17	0.68	< 0.01	0.20	< 0.01	0.53	0.77
Daily starter intake, kg	0.79	0.85	0.80	0.84	0.09	0.19	0.39	< 0.01	0.82	0.87	0.27	0.95
Average daily gain, kg	0.67^b^	0.72^a^	0.68	0.71	0.02	0.03	0.21	–	0.65	0.33	–	–
Feed efficiency^2^	0.77	0.78	0.79	0.75	0.04	0.74	0.31	–	0.30	0.68	–	–
Rectal T, °C	38.3	38.7	38.4	38.7	0.26	0.32	0.39	< 0.01	0.33	0.28	0.57	0.47
Fecal score^3^	1.83^a^	1.71^b^	1.80	1.74	0.09	0.07	0.34	< 0.01	0.67	0.09	0.53	0.13
Respiratory score^4^	1.08	1.06	1.07	1.06	0.03	0.61	0.79	0.13	0.17	0.11	0.87	0.92

^1^*M*, maternal diet effect, *C* colostrum type effect, *T* time effect.

^2^Feed efficiency (total gain/total DMI).

^3^Fecal score base on appearance: 1 = Firm well formed; 2 = Soft, pudding like; 3 = Runny, package batter; 4 = Liquid, splatters.

^4^Respiratory score base on appearance: 1 = Normal; 2 = Runny rose; 3 = Heavy breathing; 4 = Cough moist; 5 = Cough dry.

^a,b^Means on the same row differ (*P* ≤ 0.05).

### Blood plasma biomarkers at birth and during the first 50 d of age

At birth, MET calves tended to have greater cholesterol (*P* = 0.09) and NEFA (*P* = 0.07) concentrations (Table 1). Compared with CON, the concentrations of His, Lys, and Asn at birth were lower (*P* ≤ 0.05) and Met, Ala, and Tyr tended (*P* ≤ 0.10) to be lower in calves born to cows offered MET.

**Fig. 1:**
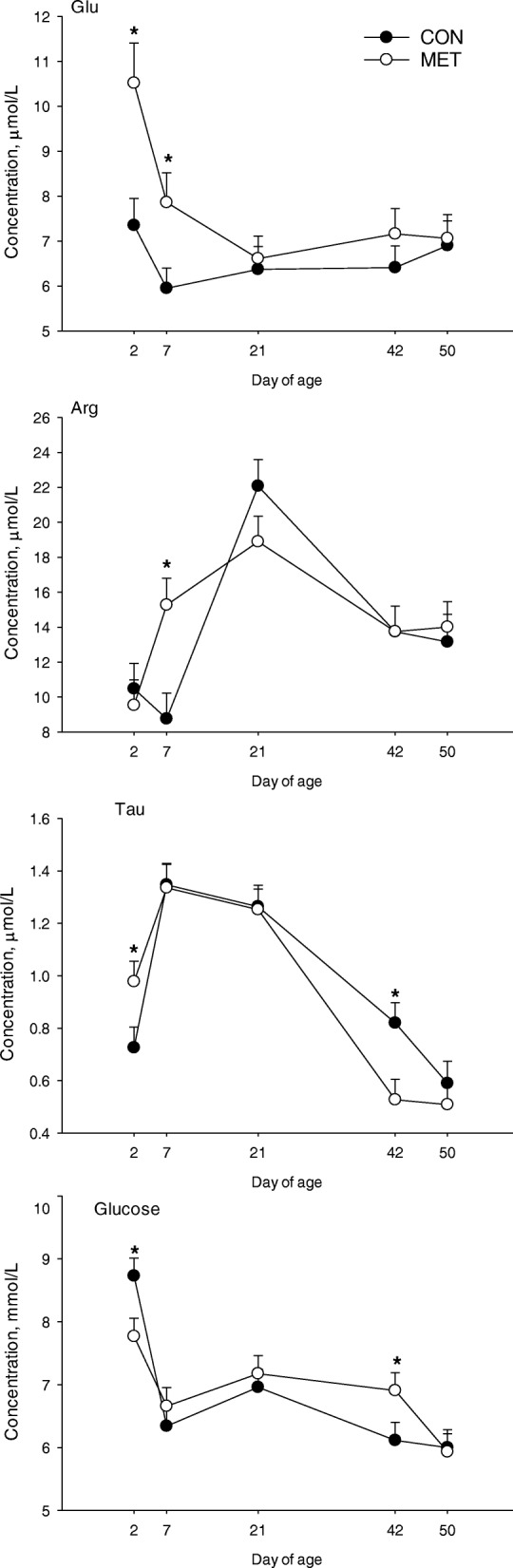
Concentrations of Glu, Arg, Tau and glucose in plasma from calves born to cows offered a control diet (CON) or CON supplemented with ethyl-cellulose rumen-protected Met (MET; Mepron® at 0.09% of diet DM; Evonik Nutrition & Care GmbH, Germany) during the last 28 d of pregnancy. Values are means, with standard errors represented by vertical bars. There was a maternal × time effect (*P* ≤ 0.05) for the concentrations of Glu, Arg, Tau and glucose. *Means between treatments differ at the same time point (*P* ≤ 0.05)

Among the biomarkers and amino acids analyzed, during the first 50 d of age, there was a main effect of maternal diet for concentrations of Glu, and Gln due to greater concentrations in MET calves (Table 3, Fig. 1). In contrast, colostrum source only affected concentration of Met which was greater (*P* = 0.04) in calves receiving MET colostrum.

**Table 3:** Plasma biomarkers and free amino acids at d 2, 7, 21, 42, and 50 of age in calves born to cows offered a control diet (CC and CM, *n* = 7/ group) or CON supplemented with ethyl-cellulose rumen-protected Met (MC and MM, *n* = 7/ group; Mepron® at 0.09% of diet DM; Evonik Nutrition & Care GmbH, Germany) during the last 28 d of pregnancy

Item	Maternal diet		Colostrum type			*P* value^1^					
	CON	MET	CON	MET	SEM	M	C	T	M × C	M × T	C × T
Biomarker, mmol/L											
Glucose	6.83	6.89	6.83	6.88	0.43	0.82	0.84	< 0.01	0.53	0.01	0.70
Cholesterol	1.86	1.97	1.90	1.94	0.12	0.30	0.69	< 0.01	0.43	0.38	0.55
NEFA	0.17	0.19	0.17	0.19	0.05	0.48	0.43	< 0.01	0.29	0.52	0.10
Urea	4.60	4.84	4.60	4.84	0.42	0.20	0.22	< 0.01	0.53	0.29	0.87
BHBA	0.11	0.13	0.11	0.12	0.04	0.06	0.21	< 0.01	0.60	0.93	0.41
Creatinine	92.6	93.8	93.3	93.1	3.14	0.68	0.91	< 0.01	0.38	0.80	0.11
Amino acids, μmol/L											
His	5.37	6.14	5.89	5.98	0.34	0.38	0.84	< 0.01	0.51	0.74	0.99
Ile	10.6	11.5	10.7	11.4	0.69	0.34	0.52	< 0.01	0.86	0.32	0.95
Leu	15.3	16.9	15.7	16.4	1.0	0.29	0.63	< 0.01	0.64	0.38	0.94
Lys	10.2	10.5	10.1	10.7	0.64	0.73	0.48	< 0.01	0.58	0.86	0.89
Met	1.95	2.10	1.85^b^	2.20^a^	0.13	0.37	0.04	< 0.01	0.03	0.44	0.43
Phe	3.99	4.02	3.92	4.09	0.19	0.91	0.56	< 0.01	0.01	0.09	0.70
Thr	11.8	11.9	12.0	11.6	0.83	0.94	0.72	< 0.01	0.95	0.76	0.97
Val	23.6	26.0	24.1	25.5	1.14	0.15	0.40	< 0.01	0.70	0.57	0.85
Ala	21.6	22.2	21.5	22.2	1.1	0.65	0.62	< 0.01	0.09	0.35	0.79
Arg	13.6	14.2	13.2	14.7	0.68	0.50	0.13	< 0.01	0.87	0.02	0.99
Asn	4.03	4.54	4.18	4.38	0.31	0.25	0.65	< 0.01	0.76	0.79	0.89
Asp	1.03	1.07	1.05	1.05	0.05	0.52	0.90	< 0.01	0.14	0.71	0.85
Glu	6.58^b^	7.73^a^	7.11	7.16	0.39	0.03	0.92	< 0.01	0.95	0.03	0.04
Gln	27.5^b^	31.2^a^	29.0	29.6	0.98	0.01	0.62	< 0.01	0.42	0.23	0.83
Gly	27.8	27.1	26.5	28.4	1.1	0.67	0.19	< 0.01	0.25	0.35	0.99
Pro	9.95	10.3	9.88	10.4	0.47	0.50	0.38	< 0.01	0.69	0.74	0.88
Ser	8.35	8.23	7.84	8.74	0.35	0.82	0.08	< 0.01	0.36	0.31	0.96
Tau	0.95	0.92	0.89	0.98	0.04	0.58	0.08	< 0.01	0.73	0.01	0.08
Tyr	4.28	4.68	4.34	4.62	0.26	0.28	0.45	0.01	0.51	0.33	0.78
Orn	0.71	0.76	0.69	0.78	0.04	0.37	0.12	< 0.01	0.43	0.35	0.78
3-methyl-His	0.06	0.05	0.06	0.05	0.01	0.52	0.86	< 0.01	0.06	0.95	0.14

^1^*M*, maternal diet effect, *C* colostrum type effect, *T* time effect.

^a,b^Means on the same row differ (*P* ≤ 0.05).

**Fig. 2:**
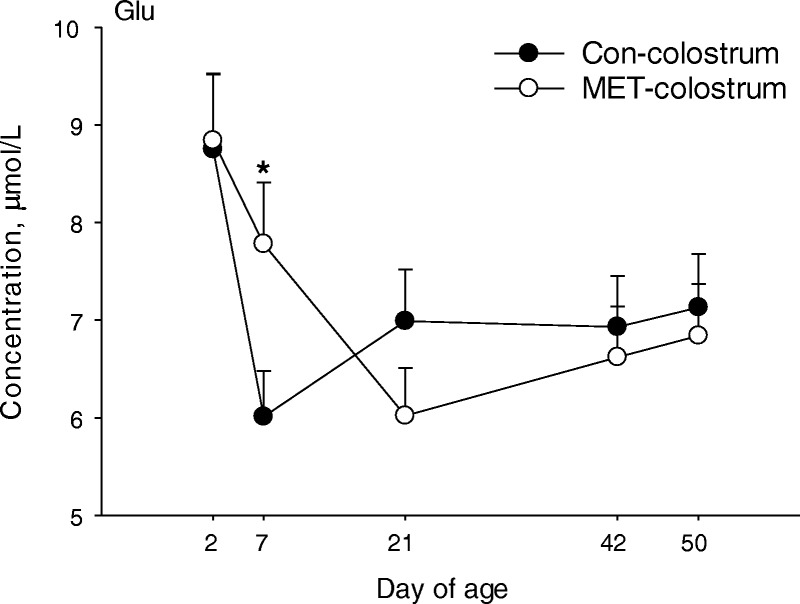
Concentrations of Glu in plasma from calves born to cows offered a control diet (CON) or CON supplemented with ethyl-cellulose rumen-protected Met (MET; Mepron® at 0.09% of diet DM; Evonik Nutrition & Care GmbH, Germany) during the last 28 d of pregnancy and fed colostrum from their respective dams. Values are means, with standard errors represented by vertical bars. There was a colostrum × time effect (*P* ≤ 0.05). *Means between treatments differ at the same time point (*P* ≤ 0.05)

**Fig. 3:**
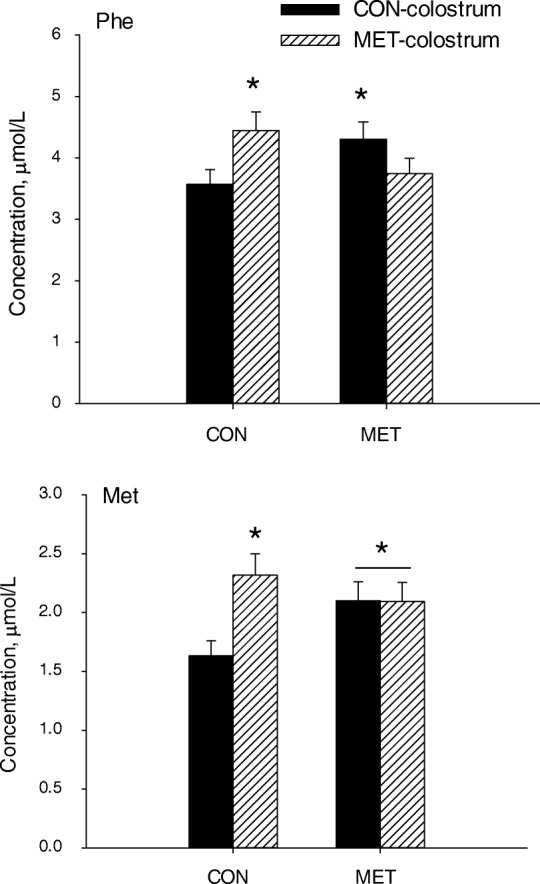
Concentrations of Met and Phe in plasma from calves born to cows offered a control diet (CON) or CON supplemented with ethyl-cellulose rumen-protected Met (MET; Mepron® at 0.09% of diet DM; Evonik Nutrition & Care GmbH, Germany) during the last 28 d of pregnancy. Approximately half of the calves in CON were fed CON colostrum (CON-CON) and the remaining calves MET colostrum (CON-MET). A similar switch was made with MET calves such that some received MET colostrum (MET-MET) or CON colostrum (MET-CON). There was a maternal × colostrum effect (*P* ≤ 0.05) for the concentrations of these amino acids. *Means between treatments differ (*P* ≤ 0.05)

Maternal × time effects (*P* < 0.01) were detected for glucose, Glu, Arg, and Tau (Table 3, Fig. 1). Calves in the MET group had greater concentrations of Glu and Tau at 2 and 7 d of age (Fig. 1). In contrast, glucose concentration was lower in MET vs. CON calves at d 2 and greater at 42 d of age (Fig. 1). A colostrum × time effect (*P* = 0.01) was detected for Glu in part due to a marked decrease in concentration from 2 to 7 d of age in calves fed CON colostrum (Fig. 2). Maternal × colostrum effects (*P* < 0.05) were detected for Met and Phe (Table 3, Fig. 3). Calves in the CON group fed MET colostrum had greater concentration of Met compared with CON calves fed CON colostrum (Fig. 3). Feeding MET colostrum to CON calves also resulted increased Phe concentrations to the same level as MET calves receiving CON colostrum and were greater than MET calves receiving MET colostrum.

### Colostrum yield, IgG concentration, amino acid, and long-chain fatty acid profiles

Colostrum IgG was not affected (*P* > 0.05) by maternal diet and averaged 52.4 ± 38.7 mg/dL for CON cows and 55.4 ± 30.8 mg/dL for MET cows. Similarly, colostrum quantity was not different between CON and MET groups (*P* > 0.05), averaging 5.7 ± 0.52 kg for CON and 5.9 ± 0.49 kg for MET cows. Concentration of Asp was greater (*P* ≤ 0.05) in colostrum from cows supplemented with MET, while crude protein and other AA were not effect by cow treatment (Additional file 1: Table S2). In terms of colostrum LCFA profile, there was a significant difference (*P* ≤ 0.05) for Methyl elaidate, Methyl 10-Transnonadecenoate, and C18:1-10 T due to greater concentrations in colostrum from cows offered MET (Additional file 1: Table S4).

## Discussion

It has been widely-recognized in recent years that environmental conditions including the maternal diet play a role in the development and growth of mammals altering the phenotype of the offspring [11, 27]. This process is usually referred as developmental programming and seems to occur during specific time points, e.g., pregnancy and/or in the pre-weaning period after birth, where certain plasticity of metabolic regulatory systems is observed [28].

In ruminants, the effect of maternal nutrition on offspring performance has been mainly explored using sheep and beef cows and the results are encouraging, emphasizing a potential area of exploration to improve animal performance. For instance, protein supplementation to beef cows during the last third of pregnancy resulted in greater calf birth BW [29]. Supplementation of RPM (6.3 g/d) during the last 41 d of pregnancy tended to increase birth BW of lambs by 10% [30]. In dairy cows, the dietary energy level during the last 21 d of pregnancy impacted calf birth BW [31] as well as height, body length, immunity and antioxidant capacity of the neonatal calves [10].

The 2 kg greater birth BW in calves from Met-supplemented cows in the present study could, at least in part, be explained by the greater prepartal daily DMI averaging + 1.2 kg/d during the last 14 d prepartum. The fact that diet can affect placental AA transport, which is dependent on both AA profiles in the maternal circulation and transport capacity [32], and the fact that feeding MET upregulated abundance of nutrient transporters (mainly glucose and AA) in placental tissue from cows in the present study [33] also underscore that there are additional mechanisms controlling nutrient delivery to the fetus. It is also noteworthy that a 1.2-kg difference in maternal DMI cannot fully account for the greater degree of difference in calf birth BW, hence, besides placental transport, efficiency of nutrient use by the fetus might have been responsible for the full effect on the calf.

Various studies have reported the effect of nutritional programming on organ development during the fetal period including muscle and small intestine [34, 35]. The number of muscle fibers is determined during the fetal period and there is little net increase after birth underscoring the importance of the fetal period on skeletal muscle [34]. Research with sheep demonstrated that, compared with controls, fetuses from ewes that were offered 50% of total digestible nutrients from d 28 to d 78 of gestation had lower numbers of myofibers [36]. In cattle, fetal skeletal muscle matures during late-gestation, hence, prenatal plane of nutrition of the cow at this time would impact muscle growth of the calf [37]. Such response explains the lower body weight at birth and muscle mass when dams are nutrient-restricted during gestation.

Although body composition was not measured in the present study, the greater concentrations of NEFA and cholesterol at birth in MET calves are suggestive of greater fat depot mass and reliance on fatty acids as primary energy source, with cholesterol potentially reflecting adaptations in lipoprotein metabolism [38]. That idea agrees with well-established knowledge that the neonate needs to activate glycogenolysis immediately after birth to maintain normal glycemia to meet its requirements for glucose [39]. In dairy calves during the first 3 d of life, 60% of the total endogenous glucose is generated through gluconeogenesis from lactate and AA [40]. Thus, the lower concentrations of His, Met, and Asn in MET calves at birth are suggestive of their utilization for endogenous glucose production.

The greater overall concentrations of Glu and Gln in MET calves were primarily associated with differences between maternal treatments at d 2 and d 7 of age, i.e. during a time when milk replacer and not starter was the primary source of nutrients for tissues including the gut. At 27% CP, milk replacer would have provided substantial amounts of Glu and Asp for metabolism, e.g. oxidation by the gastrointestinal tract [41, 42] From a physiologic standpoint, the metabolism of dietary Glu by intestinal mucosa (at least in non-ruminants) and that of arterial Gln is quite extensive particularly in actively-proliferating cells such as the neonatal jejunum and ileum [43, 44]. At least in vitro, ruminant enterocytes have the flexibility to oxidize glucose, Gln, and Glu depending on type and amount of other substrates such as acetate and propionate [45]. Thus, the fact that MET had greater concentrations of Glu and Gln during the first 7 d of life when ruminal acetate and propionate availability were likely low is suggestive that intestinal tissue might have relied on other energy-generating substrates such as glucose from lactose and long-chain fatty acids from milk replacer. This idea is reinforced by the fact that Glu oxidation by enterocytes is only reduced by 17–30% at high concentrations of propionate (10 mmol/L) or glutamine (10 mmol/L) [45].

Assuming these AA are utilized by the young calf intestine as they are in non-ruminants, the longitudinal decrease in concentrations of Glu and Gln over time regardless of maternal treatment would reflect the gradual increase in starter intake and rumen development, which agrees with the gradual increase in BHBA concentration [46]. In addition, the gradual increase over time in Asp concentration regardless of maternal Met or colostrum source could be taken as an indication of greater utilization by the developing gut (including rumen epithelium) for oxidation [47]. It remains to be determined if a “programming” effect of maternal Met had any direct role in gastrointestinal development and/or nutrient oxidation. Silencing of glutaminase via promoter hypermethylation has been demonstrated in colon cancer [48]. Thus, if such effect can occur in response to dietary methyl donors it may alter the ability of this enzyme which is abundant in gut intestinal cells to utilize Gln [42].

Although we originally sought to determine potential effects of colostrum source and quality on the post-natal growth response, the fact that it had no statistical significance for most of the outcomes measured indicated little biological effect. In the context of colostral immunoglobulins, the lack of difference in the present study agrees with other published data [49–51]. Thus, the better performance from wk 1 of birth until 9 wk of age in calves from MET-supplemented cows could have been induced by a combination of placental effects (e.g. increased nutrient supply, higher utilization efficiency) and direct fetal effects (e.g. methylation). Indeed, compared with lambs born to dams consuming dietary energy at 80% metabolizable energy (ME), maternal dietary energy at 120% of ME led to greater ADG from birth to weaning [52]. Those data agree with studies from non-ruminant animals reporting that birth weight is positively correlated with ADG [53, 54]. Along with greater ADG, the fact that BW, HH, and WH of MET calves was not only greater at birth but through 56 d of age suggests that the mechanisms responsible were likely programmed in utero rather than after birth.

In non-ruminants, the maternal supplementation of methyl donors affects the offspring; the supplementation of betaine [55] and folic acid [56] to pregnant sows increased protein abundance of gluconeogenic enzymes and of proteins that regulate the immune response and energy metabolism in the neonatal piglet. Although knowledge of the impacts of methyl donor supply in dairy cows is still in its infancy, an increase in maternal Met supply during the last 21 d of pregnancy upregulated the expression of genes involved in gluconeogenesis and fatty acid oxidation in neonatal calf liver, which could benefit the calf’s adaptations to extrauterine life and subsequent growth [19].

The longer-term carryover effects of maternal supply of Met and other methyl donors on the offspring could be due to the link between epigenetic mechanisms and one carbon metabolism. The latter integrates folate and Met cycles and generates SAM [57], which impacts DNA methylation and can elicit epigenetic alterations that often lead to silencing of gene transcription [58]. Histone methylation is another epigenetic mechanism and it performs diverse functions in the establishment of the chromatin states (euchromatin or heterochromatin) that mediate the regulation of gene expression [57]. The activities of histone methyltransferase are also dependent on intracellular levels of SAM [59]. Presently, there is not much information on the role of methyl donor availability on epigenetic mechanisms linked with fetal programing in beef and dairy cattle. Hence, it is necessary to identify how the epigenome impacts physiologic responses and how specific nutrients or dietary interventions in utero could modulate animal production through epigenomic alterations.

## Conclusions

Overall, our findings provide evidence that enhancing the supply of Met during the last 28 d of pregnancy not only led to increased growth of the calf in utero, but altered mechanisms regulating postnatal growth such that differences detected at birth remained through the early post-weaning period. At the whole-animal level these effects did not seem to be completely associated with prepartal dry matter intake differences or colostrum quality. Thus, utero-placental effects specific to the greater supply of Met (and potentially other amino acids) likely played a mechanistic role. Clearly, additional research in this area to clarify the underlying mechanisms is warranted.

## Additional file

Additional file 1: **Table S1.** Ingredient and nutrient composition of diets fed to cows. **Table S2.** Amino acid profiles and crude protein content of colostrum. **Table S3.** Amino acid profiles of milk replacer and starter grain. **Table S4.** Fatty acid profiles of colostrum. **Table S5.** Effects of supplementing Holstein cows during the peripartal period with rumen-protected methionine (MET; Mepron®, Evonik Nutrition & Care GmbH, Germany) on colostrum fatty acid profile. **Figure S1.** Body weight, hip height, and wither height during the first 9 wk of life, and daily starter intake (1–56 d of age) in calves born to cows offered control diet (CON) or CON supplemented with ethyl-cellulose rumen-protected Met (MET; Mepron® at 0.09% of diet DM; Evonik Nutrition & Care GmbH, Germany) during the last 28 d of pregnancy. (DOCX 61 kb)

## Abbreviations

AA: Amino acids
ADG: Average daily gain
BHBA: Hydroxybutyric acid
BW: Body weight
CC: Calves from CON cows and colostrum from CON cows
CM: Calves from CON cows and colostrum from MET cows
CON: Control-fed cows
CP: Crude protein
DM: Dry matter
EAA: Essential amino acids
HH: Hip height
HW: Hip width
IgG: Immunoglobulin G
LCFA: Long-chain fatty acids
MC: Calves from MET cows and colostrum from CON cows
ME: Metabolizable energy
MET: Cows fed rumen-protected Met
MM: Calves from MET cows and colostrum from MET cows
NDF: Neutral detergent fiber
NEFA: Free fatty acids
RPM: Rumen-protected Met
SAM: S-adenosylmethionine
WH: Wither height
