# Supplementary material for: Maternal supply of methionine during late-pregnancy enhances rate of Holstein calf development in utero and postnatal growth to a greater extent than colostrum source
Source: J Anim Sci Biotechnol. 2018 Nov 23;9:83. doi: 10.1186/s40104-018-0298-1 (PMC6251175; doi:10.1186/s40104-018-0298-1)
Supplement: Supplementary file 1 — Table S1. Ingredient and nutrient composition of diets fed to cows. Table S2. Amino acid profiles and crude protein content of colostrum. Table S3. Amino acid profiles of milk replacer and starter grain. Table S4. Fatty acid profiles of colostrum. Table S5. Effects of supplementing Holstein cows during the peripartal period with rumen-protected methionine (MET; Mepron®, Evonik Nutrition & Care GmbH, Germany) on colostrum fatty acid profile. Figure S1. Body weight, hip height, and wither height during the first 9 wk of life, and daily starter intake (1–56 d of age) in calves born to cows offered control diet (CON) or CON supplemented with ethyl-cellulose rumen-protected Met (MET; Mepron® at 0.09% of diet DM; Evonik Nutrition & Care GmbH, Germany) during the last 28 d of pregnancy. (DOCX 61 kb) [file 40104_2018_298_MOESM1_ESM.docx]

**Table S1.** Ingredient and nutrient composition of far-off (from −45 to −29 d) and close-up (from −28 d to parturition) diets fed to cows.

| Ingredient, % of DM | Diets | |
| --- | --- | --- |
|  | Far-off | Close-up |
| Alfalfa haylage | ­­ — | 6.55 |
| Corn silage | 34.7 | 26.6 |
| Wheat straw | 33.7 | 26.5 |
| Corn grain, ground, dry | — | 12.6 |
| Molasses, beet sugar | — | 4.03 |
| Soybean hulls | 15.7 | 3.46 |
| Soybean meal, 48% CP | 12.0 | 7.83 |
| Expeller soybean meal^1^ | — | 5.80 |
| Protein supplement^2^ | — | 0.78 |
| Urea | 0.46 | 0.59 |
| Soychlor^3^ | — | 1.23 |
| Salt | 0.40 | — |
| Dicalcium phosphate | 0.50 | 0.52 |
| Magnesium sulfate | 1.90 | 2.08 |
| Mineral vitamin mix^4^ | 0.40 | 0.17 |
| Vitamin A^5^ | — | 0.03 |
| Vitamin D^6^ | — | 0.03 |
| Vitamin E^7^ | 0.40 | 0.60 |
| Biotin^8^ | — | 0.70 |
| Monensin^9^ | 0.01 | — |
| Ethyl-cellulose rumen-protected Met^10^ | — | 0.09 |

^1^SoyPlus, West Central Soy (Ralston, IA).

^2^ProVAAl AADvantage, Perdue AgriBusiness (Salisbury, MD).

^3^West Central Soy.

^4^Contained a minimum of 5% Mg, 10% S, 7.5% K, 2.0% Fe, 3.0% Zn, 3.0% Mn, 5,000 mg of Cu/kg, 250 mg of I/kg, 40 mg of Co/kg, 150 mg of Se/kg, 2,200 kIU of vitamin A/kg, 660 kIU of vitamin D3/kg, and 7,700 IU of vitamin E/kg.

^5^Contained 30,000 kIU/kg.

^6^Contained 5,000 kIU/kg.

^7^Contained 44,000 kIU/kg.

^8^ADM Animal Nutrition (Quincy, IL).

^9^Rumensin, Elanco Animal Health (Greenfield, IN).

^10^Evonik Nutrition and Care GmbH (Hanau-Wolfgang, Germany). Added only in the treatment group.

**Tables S2.** Energy, metabolizable protein (MP), and post-ruminal Lysine (Lys) and Methionine (Met) availability in response to feeding a far-off (from −45 to −29 d) and close-up (from −28 d to parturition) diet. The NRC (2001)^1^ evaluation of diets was based on final averaged prepartum dry matter intake and feed analysis.

|  | Diets | | |
| --- | --- | --- | --- |
|  |  | Close-up | |
| Item | Far-off | Control | Methionine |
| NE_L_, Mcal/kg DM | 1.33 | 1.47 | 1.47 |
| MP required, g/d | 790 | 808 | 808 |
| MP supplied, g/d | 1,211 | 1,363 | 1,473 |
| MP balance, g/d | 421 | 555 | 664 |
| Lys, % of MP | 6.74 | 6.54 | 6.51 |
| MP-Lys, g | 82 | 89 | 89 |
| Met, % of MP | 1.77 | 1.73 | 2.30 |
| MP-Met, g | 21 | 24 | 32 |
| Lys:Met | 3.81:1 | 3.71:1 | 2.81:1 |

^1^NRC. Nutrient requirements of dairy cattle. 7th rev. ed. Washington, D.C.: National Academy Press; 2001.

**Table S3.** Effects of supplementing Holstein cows during the peripartal period with rumen-protected methionine (MET; Mepron^®^, Evonik Nutrition & Care GmbH, Germany) on colostrum amino acid content.

|  | Treatment | | SEM |  |
| --- | --- | --- | --- | --- |
|  | CON | MET |  | *P*-value |
| Crude protein, % of DM | 72.69 | 72.94 | 0.76 | 0.82 |
| Indispensable AA, % of DM | |  |  |  |
| Arginine | 3.14 | 3.19 | 0.05 | 0.44 |
| Histidine | 1.87 | 1.90 | 0.02 | 0.32 |
| Isoleucine | 2.98 | 2.97 | 0.02 | 0.82 |
| Leucine | 6.58 | 6.70 | 0.07 | 0.26 |
| Lysine | 5.56 | 5.65 | 0.05 | 0.23 |
| Methionine | 1.33 | 1.35 | 0.02 | 0.38 |
| Phenylalanine | 3.28 | 3.22 | 0.03 | 0.20 |
| Threonine | 4.87 | 5.04 | 0.09 | 0.18 |
| Valine | 5.27 | 5.37 | 0.07 | 0.28 |
| Dispensable AA, % of DM | |  |  |  |
| Aspartate | 6.20 | 6.43 | 0.08 | 0.04 |
| Alanine | 2.87 | 2.91 | 0.04 | 0.45 |
| Cysteine | 1.38 | 1.42 | 0.03 | 0.43 |
| Glutamate | 11.7 | 11.9 | 0.11 | 0.36 |
| Glycine | 2.29 | 2.39 | 0.21 | 0.72 |
| Proline | 6.07 | 6.13 | 0.06 | 0.46 |
| Serine | 5.70 | 5.92 | 0.11 | 0.15 |

**Table S4.** Amino acid profiles in milk replacer and calf starter fed to calves to evaluate the effect of rumen-protected methionine (MET; Mepron^®^, Evonik Nutrition & Care GmbH, Germany) supplementation of Holstein cows during pregnancy on calf growth.

| Item | Starter | | Replacer | |
| --- | --- | --- | --- | --- |
|  | Mean | SD | Mean | SD |
| Dry matter, g/kg | 908 | 27 | 946 | 11 |
| Crude protein, g/kg | 209 | 23 | 272 | 44 |
| Essential AA, % of DM |  |  |  |  |
| Arginine | 1.39 | 0.03 | 0.84 | 0.07 |
| Histidine | 0.55 | 0.01 | 0.59 | 0.02 |
| Isoleucine | 0.82 | 0.01 | 1.60 | 0.06 |
| Leucine | 1.52 | 0.03 | 2.90 | 0.07 |
| Lysine | 1.07 | 0.02 | 2.30 | 0.12 |
| Methionine | 0.29 | 0.01 | 0.53 | 0.02 |
| Phenylalanine | 0.98 | 0.01 | 1.01 | 0.02 |
| Threonine | 0.77 | 0.02 | 1.83 | 0.04 |
| Valine | 0.97 | 0.01 | 1.59 | 0.03 |
| Non-essential AA, % of DM |  |  |  |  |
| Aspartate | 2.00 | 0.05 | 2.89 | 0.07 |
| Alanine | 0.97 | 0.02 | 1.36 | 0.03 |
| Cysteine | 0.34 | 0.01 | 0.64 | 0.02 |
| Glutamate | 1.07 | 0.02 | 4.68 | 0.15 |
| Glycine | 0.98 | 0.01 | 0.60 | 0.03 |
| Proline | 1.14 | 0.02 | 1.64 | 0.04 |
| Serine | 0.99 | 0.03 | 1.47 | 0.05 |
| NH_3_ | 0.77 | 0.02 | 0.53 | 0.01 |

**Table S5.** Effects of supplementing Holstein cows during the peripartal period with rumen-protected methionine (MET; Mepron^®^, Evonik Nutrition & Care GmbH, Germany) on colostrum fatty acid profile.

|  | Treatment | | SEM |  |
| --- | --- | --- | --- | --- |
| Fatty acid, % of total fatty acids | CON | MET |  | *P*-value |
| C6:0 Methyl Caproate | 0.44 | 0.42 | 0.02 | 0.44 |
| C8:0 Methyl Caprylate | 0.36 | 0.34 | 0.02 | 0.56 |
| C10:0 Methyl Decanoate | 1.06 | 0.96 | 0.07 | 0.34 |
| C12:0 Methyl Laurate | 2.03 | 1.81 | 0.14 | 0.27 |
| C14:0 Methyl Myristate | 11.1 | 10.4 | 0.66 | 0.47 |
| C14:1T Methyl Transmyristelaidate | 0.14 | 0.12 | 0.01 | 0.17 |
| C14:1 Methyl Myristoleate | 0.49 | 0.45 | 0.02 | 0.20 |
| C15:0 Methyl Pentadecanoate | 0.77 | 0.75 | 0.08 | 0.82 |
| C16:0 Methyl Palmitate | 42.0 | 42.3 | 1.30 | 0.86 |
| C16:1T Methyl Palmitelaidate | 0.20 | 0.20 | 0.01 | 0.92 |
| C16:1 Methyl Palmitoleate | 2.21 | 2.20 | 0.12 | 0.92 |
| C17:0 Methyl Heptadecanoate | 0.60 | 0.60 | 0.02 | 0.99 |
| C17:1T Methyl 10-Heptadecenoate | 0.53 | 0.53 | 0.05 | 0.95 |
| C18:0 Methyl Stearate | 7.69 | 7.78 | 0.50 | 0.90 |
| C18:1-9T Methyl elaidate | 0.11 | 0.14 | 0.01 | 0.02 |
| C18:1-10T | 0.13 | 0.15 | 0.01 | 0.02 |
| C18:1-11T Methyl Transvaccenate | 0.57 | 0.59 | 0.03 | 0.66 |
| C18:1Methyl Oleate | 23.9 | 24.7 | 1.45 | 0.69 |
| C18:1-11C Methyl Vaccinate | 0.75 | 0.82 | 0.05 | 0.34 |
| C18:2T Methyl Linoelaidate | 0.25 | 0.29 | 0.02 | 0.13 |
| C19:1T Methyl 10-Transnonadecenoate | 0.12 | 0.15 | 0.01 | 0.03 |
| C20:0 Methyl Arachidate | 0.04 | 0.04 | 0.003 | 0.70 |
| C18:2 Methyl Linoleate | 2.88 | 3.01 | 0.10 | 0.34 |
| C18:3 Methyl Alpha Linolenate | 0.21 | 0.22 | 0.01 | 0.40 |
| C22:0 Methyl Behenate | 0.06 | 0.06 | 0.004 | 0.94 |
| C18:2-cis-9, trans-11Methyl Rumenate | 0.19 | 0.19 | 0.01 | 0.43 |
| C23:0 Methyl Tricosanoate | 0.27 | 0.31 | 0.02 | 0.11 |
| C20:4 Methyl Arachidonate | 0.44 | 0.47 | 0.02 | 0.35 |
| C20:5 Methyl Eicosapentaenoate | 0.05 | 0.06 | 0.003 | 0.33 |
| C24:1 Methyl Nervonate | 0.08 | 0.09 | 0.004 | 0.28 |
| C22:5N3 Methyl Docosapentaenoate | 0.13 | 0.14 | 0.01 | 0.33 |
| Total content, mg/g | 591 | 619 | 13 | 0.14 |

**Figure S1.** Body weight, hip height, and wither height during the first 9 weeks of life, and daily starter intake (1-56 d of age) in calves born to cows offered control diet (CON) or CON supplemented with ethyl-cellulose rumen-protected Met (MET; Mepron^®^ at 0.09% of diet DM; Evonik Nutrition & Care GmbH, Germany) during the last 28 d of pregnancy. There was a maternal effect (*P* ≤ 0.05) for body weight, hip height, and wither height. There was a time effect (*P* ≤ 0.05) for daily starter intake
